# Exploring the Mechanisms and Association between Oral Microflora and Systemic Diseases

**DOI:** 10.3390/diagnostics12112800

**Published:** 2022-11-15

**Authors:** Rakhi Issrani, Jagat Reddy, Tarek H. El-Metwally Dabah, Namdeo Prabhu, Mohammed Katib Alruwaili, Manay Srinivas Munisekhar, Sultan Meteb Alshammari, Shmoukh Fahad Alghumaiz

**Affiliations:** 1 Department of Preventive Dentistry, College of Dentistry, Jouf University, Sakaka 72388, Saudi Arabia; 2 Oral Medicine & Radiology, Indira Gandhi Institute of Dental Sciences, SBV University, Puducherry 607402, India; 3 Department of Pathology, Medical Biochemistry Division, College of Medicine, Jouf University, Sakaka 72388, Saudi Arabia; 4 Department of Medical Biochemistry, Faculty of Medicine, Assiut University, Assiut 71527, Egypt; 5 Department of Oral & Maxillofacial Surgery and Diagnostic Sciences, College of Dentistry, Jouf University, Sakaka 72388, Saudi Arabia; 6 Department of Periodontology and Endodontology, Division of Oral Health Science, Faculty of Dental Medicine, Hokkaido University, Sapporo 060-0808, Hokkaido, Japan

**Keywords:** bacteria, diseases, pathogens, periodontitis, systemic

## Abstract

The scope of dentistry is ever-changing and dynamic in all fields of dentistry including periodontal health and disease. Recent studies show that oral health and systemic health are interdependent, particularly in the way that poor oral hygiene and periodontal health affect the systemic health of an individual and vice versa. Periodontal diseases are multifactorial in nature in which the role of bacterial infections is inevitable. Furthermore, high-throughput sequencing technologies have shed light on the dysregulation of the growth of oral microbial flora and their environment, including those that are associated with periodontitis and other oral and non-oral diseases. Under such circumstances, it becomes important to explore oral microbiota and understand the effects of periodontal pathogens in the pathogenesis of systemic diseases. In addition, it may strengthen our view that a better understanding of oral microbial flora and proper examination of the oral cavity may aid in the early diagnosis and possible treatment of systemic diseases and conditions. This will eventually lead to providing better care to our patients. Therefore, in this research, we attempt to outline the periodontal pathophysiology along with the role of periodontal pathogens in some commonly encountered systemic conditions.

## 1. Introduction

The oral cavity serves as a good medium for bacterial growth and it harbors a variety of microorganisms with approximately 500–700 prevalent taxa [1]. This microbial community is known as the oral microbiota, oral microflora, or oral microbiome [2]. However, its composition varies constantly throughout its life which is determined by a host of factors such as oral hygiene, dietary habits, the use of medications, host genetics, presence of exogenous microorganisms and some systemic factors [3]. With the advancements in isolation and culture techniques and the availability of more specific culture media, a better understanding of the coexistence of such complex polymicrobial communities as well as the role of local and systemic factors that govern/dictate the composition of oral microbiota was made possible [4].

For over a century, although it was assumed that oral diseases and systemic diseases were interrelated, there was no adequate scientific evidence [3]. However, as late as the 1980s and 1990s, a concerted effort was made to gather scientific evidence to strengthen such opinion [5,6]. Subsequently, a lot of research was carried out to investigate and analyze the causal role of periodontal disease and certain systemic diseases such as diabetes, atherosclerotic cardiovascular disease and adverse pregnancy outcomes [7,8,9,10]. Periodontitis and its association with a variety of other diseases such as obesity, diabetes, rheumatoid arthritis, impairment of cognition, cancer, metabolic syndrome and respiratory disease have also been reported [1,10,11]. Presently, it is well-established that oral health or disease and systemic health or disease are strongly interrelated and opened new avenues for research to investigate and identify the presence of biomarkers in the oral cavity which could help in the early diagnosis of and an appropriate treatment for the systemic diseases. Thus, the oral cavity could serve as a non-invasive tool for diagnosis and prevention of systemic diseases and their successful treatment for a healthier socioclinical scenario.

Therefore, in this paper, an attempt is made to outline the periodontal pathophysiology along with the role of periodontal pathogens in some commonly encountered systemic conditions.

## 2. Periodontal Pathophysiology

In adults, periodontal disease is a common inflammatory disease affecting nearly 3.9 billion people worldwide with 35% having mild periodontitis and 11% having moderate to severe periodontitis [12]. With the aging population, this disease has become a significant public health concern and also a mounting burden on the healthcare system [13]. The US Centers for Disease Control and Prevention suggested that it is a pandemic affecting all geographic locations across the globe resulting in loss of teeth with resultant pain and malnutrition, impairment of speech and mastication leading to a poor quality of life for the patient [10,13].

Its pathogenesis is governed by the complicated and constant interactions between the related pathogens and the immune system. In addition to the direct effect of the main etiology of ‘pathogenic bacteria and its byproduct in a susceptible host’, knowledge and understanding of periodontitis and various factors such as genetics and immune reactions of the host have improved over the years due to extensive research in this area [1].

Based on their pathogenicity, the periodontal pathogens are grouped into complexes. Amongst them, the red complex is the most pathogenic and includes *Porphyromonas gingivalis* (*P. gingivalis*); *Treponema denticola* (*T. denticola*); and *Tannerella forsythia* (*T*. *forsythia*) [2]. In addition, there is increasing evidence that certain Gram-negative bacteria such as *Aggregatibacter actinomycetemcomitans* (*A. actinomycetemcomitans*) and *Fusobacterium nucleatum* (*F. nucleatum*) and certain Gram-positive *Streptococcus* spp and *Filifactor alocis* also play an important role in the etiopathogenesis of periodontitis [9]. These putative pathogens are said to possess a variety of virulence factors that regulate the initiation of disease and its progression [9]. 

### Pathogenesis

The following two mechanisms explain the role of periodontal disease in systemic diseases:

*a*. Direct mechanism: The endotoxins produced mainly by Gram-negative anaerobic bacteria in the oral cavity are known to directly contribute to systemic disease. The periodontal pathogens gain access into the blood circulation through the ulcers located in the soft tissue walls of the periodontal pockets resulting in collagen degradation, aggregation of platelets and thrombus formation [14,15,16];
*b*. Indirect mechanism: This involves a possible trigger to periodontal pathogens producing inflammatory mediators in the body such as C-reactive protein (CRP), tumor necrosis factor (TNF)-α, interleukin (IL)-1α, IL-1β, IL-6, prostaglandin E2 (PGE2) and matrix metalloproteinases (MMP) causing an autoimmune reaction [10,17].

Thus, the biological connection between periodontitis and systemic conditions could be explained based on the following facts [10]:

(a) The usual implication of infection in the pathogenesis of both diseases;
(b) Transient and low grade bacteremia and endotoxemia caused by periodontal diseases;
(c) Expression of virulence factors by periodontal pathogens;
(d) Inflammation and systemic immune responses triggered by periodontal diseases;
(e) Presence of periodontal pathogens in non-oral tissues such as atheromatous plaques.

Although the exact mechanisms for such a process are not clear, enough scientific data are available to suggest that such a bilateral association exists between the systemic and periodontal diseases and that they could aggravate each other [1,10].

## 3. Periodontitis and Systemic Diseases

Periodontitis has been identified as an important predisposing factor for many systemic diseases [9]. In this section, its role in the development and progression of few systemic diseases is discussed. 

### 3.1. Cardiovascular Diseases (CVD)

Congestive heart failure, cardiac arrhythmias, valvular heart disease, coronary artery disease and stroke constitute CVD. Atherosclerosis plays a very dominant role in the etiopathogenesis of CVD [18]. It is presumably one of the most important causes of death across the world [19]. Extensive research was carried out to establish a possible link between periodontal disease and CVD.

Both periodontitis and CVD are similar in that they are chronic processes and multifactorial in nature and certain risk factors such as age and gender, smoking, stress and lower socioeconomic status [20]. Studies have also shown that individuals with periodontitis had an increased risk of developing coronary heart disease as compared to the control group, independent of the other precipitating factors [5,21,22]. Microbiological studies of human atherosclerotic plaques have shown the presence of a variety of periodontal pathogens such as *T. forsythia*, *A. actinomycetemcomitans*, *P. gingivalis* and *Prevotella intermedia* (*P. intermedia*), indicating that these organisms reach distant sites from the oral cavity [23,24,25,26]. Further, oral streptococci, which constitute a part of dental plaque, is also found as a causative factor to vulnerable surfaces in the cardiovascular system such as damaged endocardium and can provoke bacterial endocarditis [1]. Additionally, *Chlamydia pneumoniae* (*C. pneumoniae*) and certain other bacteria are also implicated as a risk factor in the development of myocardial infarction and hypertension [27,28].

More recent studies have suggested that oral infection with bacteria such as *P. gingivalis* and *T. denticola* initiate a systemic immune response [29,30]. It was also shown that *P. gingivalis* escapes detection by Toll-like receptor-4 (TLR-4) and facilitates a chronic inflammatory process in the blood vessels [31]. According to an in vitro study by Herzberg et al., it was also shown that *P. gingivalis* has factors that can potentially induce the aggregation of human platelets and thus it could possibly be responsible for the formation of thrombus in vivo [32]. On the contrary, other periodontal pathogens such as *Campylobacter rectus* (*C. rectus*), *A. actinomycetemcomitans*, *F. nucleatum*, *T. forsythia*, *T. denticola* and *P. intermedia* could not induce such a process, indicating that *P. gingivalis* expressed virulent factors responsible for induction of platelet aggregation [33].

Presently, it is also not clear if periodontal treatment at an appropriate time would minimize the chance of developing the adverse cardiovascular events. However, it would be interesting to dwell on this area to assess if such cardiovascular events can be prevented by instituting early periodontal therapy. Though some studies have suggested the effects of periodontal therapy on CRP levels which in turn are associated with CVD, a recent systematic review did not support the view that there would be a significant reduction in the levels of CRP following periodontal therapy [34,35]. 

### 3.2. Obesity, Metabolic Syndromes, and Diabetes Mellitus

Obesity is considered as a chronic disease which is associated with a variety of diseases such as type 2 diabetes, coronary heart disease, hypertension and cancer [36]. However, it is also said to have a negative impact on periodontal health [37]. The increased release of pro-inflammatory cytokines in the adipose tissue is thought to initiate certain processes that result in such associations [38].

Metabolic syndrome constitutes a group of metabolic disturbances that are associated with an increased risk of diabetes 2 and CVD. Cross-sectional studies have shown that metabolic syndrome is more likely to develop in individuals with moderate to severe periodontitis than those individuals without, or with mild periodontitis [39]. A cohort study has shown that deeper probing depths and greater loss of alveolar bone were related to chronic metabolic syndrome [40]. There might be decreased insulin sensitivity, reduced antioxidant capacity and enhanced oxidative damage which may build up the oxidative stress and act as a bidirectional link between the metabolic syndrome and periodontitis [41]. 

However, studies regarding specific periodontal pathogens neither associated with obesity nor metabolic syndrome are available.

Diabetes mellitus is a metabolic disturbance associated with hyperglycemia due to disturbances in insulin secretion and its action or both [41]. Uncontrolled diabetes is associated with severe periodontitis which is considered to be sixth most common complication of the disease [42]. A “bidirectional relationship” exists between hyperglycemia and the severity of periodontitis. Studies have also shown that patients with diabetes are 2.8 times more prone to develop destructive periodontal disease [43] and 4.2 times more prone to developing progressive alveolar bone loss as compared to those without it [44].

Periodontal pathogens such as *A. actinomycetemcomitans*, *Eikenella corrodens* (*E. corrodens*), *T. denticola*, *Candida albicans*, and *P. gingivalis* have been shown to be the most prevalent bacteria in patients with diabetes. In such individuals, these bacteria may aggravate a variety of microvascular complications such as nephropathy, retinopathy and neuropathy which in turn may result in macrovascular complications such as CVD, coronary artery disease and peripheral vascular disease. Studies have shown that diabetic individuals with severe periodontitis are three times more prone to end-stage renal disease and two times more prone to macroalbuminuria and cardiorenal mortality [1]. 

Chronic systemic inflammation induced by the periodontal pathogens is thought to be an underlying mechanism that links these two entities [45]. Periodontal infection can further intensify the state of existing systemic inflammation and increase insulin resistance. Experimental studies on diabetic rats showed that with infection with *P. gingivalis* there was reduced gingival vascular function and an increase in insulin resistance [46].

Many studies have proved that there exists a synergism between periodontitis and diabetes and that institution of an appropriate and effective periodontal therapy helps in achieving better control of blood-sugar levels, particularly in type 2 diabetes, and low glycated hemoglobin levels [47,48]. Both diabetic and non-diabetic patients respond well to periodontal therapy. On the contrary, in uncontrolled diabetes, the recurrence of periodontal disease, more particularly a severe form with extensive destruction of periodontal tissues is expected [49].

### 3.3. Adverse Pregnancy Outcomes (APO)

Over the last two decades, extensive research was focused on studying the association between APO and periodontal diseases. It was found that pregnant women with active periodontitis were at a greater risk of developing a variety of APO such as gestational diabetes, pre-eclampsia, fetal growth restriction, preterm birth and low birth weight [50]. A case-control study showed that poor maternal periodontal status was strongly associated with preterm, low-birth weight babies [51]. 

Many possible direct and indirect mechanisms have been suggested to describe the underlying pathophysiological process of active maternal periodontitis leading to APO [52]. In the direct mechanism, there is a vertical transmission of periodontal pathogens into the placenta through the umbilical cord leading to its dysfunction [53]. However, in the indirect mechanism, due to the release of certain pro-inflammatory cytokines into the systemic circulation there is a local inflammatory response at the fetoplacental unit. Due to periodontitis, cytokines such as TNF-α, IL-8, IL6 and IL-1 are released which activate a cascade of events resulting in the release of excessive PGE2 which in turn leads to APO through uterine contractions [52].

The most prevalent periodontal pathogen in placental and fetal tissues is *F. nucleatum* [54]. It becomes translocated into the uterus when the immune system of the mother is compromised and results in stillbirth [55]. It is frequently isolated from cord blood and amniotic fluid in preterm born babies and in neonatal sepsis [56,57] and in certain intrauterine infections suggesting translocation from the oral tissues [54,57]. 

Studies have shown the presence of *P. gingivalis* and its endotoxins within the placental tissues in preterm babies [53,58]. Experimental studies on rats have shown that lipopolysaccharide from *P. gingivalis* initiated a restriction of placental and fetal growth and resorption, suggesting that they have a negative impact on pregnancy [59]; additionally, when antibodies against *P. gingivalis* were identified, there was fetal loss [60]. The maternal–fetal interface is a unique site immunologically that favors fetal immune tolerance and simultaneously wards off the chances of any possible infection. Although the exact role of the innate immune receptors in pregnancy is still obscure, studies have shown that placenta expresses TLRs during the prenatal development of the fetus [61]. It was shown that there is an increased placental expression of TLRs in the presence of *P. gingivalis* and *T. denticola*, suggesting an active innate response [62,63]. Although the existing data indicate that all preventive measures should be ensured in pregnant women against periodontitis, more studies may be necessary to strengthen the cause-and-effect relationship between the two diseases. 

### 3.4. Respiratory Tract Infection

Respiratory infections such as chronic obstructive pulmonary diseases (COPD) and pneumonia may result from the hematogenous spread or aspiration of periodontal pathogens to the upper and lower airway [10]. This could be either due to a compromised immune response of the host resulting in the aspiration of associated pathogens from the oral cavity or it could be due to the release of virulence factors (enzymes and cytokines) by the periodontal pathogens which in turn initiate an inflammatory process within the lungs. Generally, the respiratory tract is competent to defend against these bacteria and their toxins in healthy individuals [10]. However, a variety of factors and disabilities such as reduced rate of salivary flow, poor oral hygiene, dysphagia and diminished cough reflex render the individual susceptible to pulmonary infections [10]. 

A variety of periodontal pathogens such as *Streptococcus constellatus*, *E. corrodens*, *P. intermedia*, *P. gingivalis*, *Actinomyces israelii*, *A. actinomycetemcomitans*, *Capnocytophaga* spp., *C. pneumoniae*, *F. necrophorum* and *F. nucleatum* have been implicated in the etiopathogenesis of airway infections [64,65,66]. Genetic constitution of bacteria isolated from both bronchoalveolar lavage fluid and dental plaque were shown to be similar, strengthening the view that unchecked periodontitis may lead to respiratory infections [67]. *P. gingivalis* was also shown to be involved in the persistent inflammatory responses within the lungs through constant recruitment of the cells and cytokines [64]. Interestingly, in both edentulous and dentulous patients who were orotracheally intubated, periodontal pathogens such as *T. forsythia*, *P. gingivalis* and *A. actinomycetemcomitans* were found in substantial quantities, suggesting that the oral cavity serves as a potential medium for growth and accumulation of pathogenic bacteria [68]. Certain common periodontal pathogens such as *F. necrophorum* and *F. nucleatum* were involved in the causation of a specific respiratory tract infection, which begins with pharyngitis and ultimately culminates in Lemierre’s syndrome [65,66]. Studies have also shown that *F. necrophorum* was twice more common than group A b-hemolytic streptococcus in sore throats and asymptomatic individuals, suggesting that it is a potential pathogen of lungs and should be investigated during airway complications [69]. On the contrary, *C. pneumoniae* is a well-established respiratory pathogen that has been linked to COPD, bronchitis and asthma [70]. It is commonly seen in the oral cavity and probably gets translocated to the lower airways, from where it spreads to distant sites such as the aorta, heart and spleen by monocytes through hematogenous route [71,72,73]. In addition, it has been related to an enhanced risk of atherosclerosis development suggesting that it could be another possible mechanism by which periodontal pathogens lead to atherosclerosis [74].

Randomized controlled studies have shown that improved oral hygiene plays a significant role in preventing pneumonia in high-risk populations. 

### 3.5. Cancer

The human microbiome has been unequivocally linked to cancer. Cancers caused by human papilloma virus, helicobacter pylori and Hepatitis B and C infections constitute about 15% of cancers globally [1].

A variety of cancers affecting the oral cavity, gastrointestinal tract, prostate gland, breast, lung and uterus have been linked to periodontal disease [1,10,75]. While a significantly high level of *P. gingivalis* was demonstrated in oro-esophageal carcinoma [76], Gram-negative anaerobic bacteria such as *Campylobacter*, *Leptotrichia* and *Fusobacterium* species were found in colorectal cancers in large quantities [77]. It was shown that *F. nucleatum* migrated to the gastrointestinal tract and caused inflammatory infections [1]. In addition, when the role of *F. nucleatum* was assessed for its possible role in intestinal tumorigenesis, it was associated with an increase in tumor multiplicity and promoted tumor progression [1]. In another study, a large number of *F. nucleatum* were found in human mucosal and fecal samples in colorectal adenoma patients [1]. 

With regard to periodontitis and breast cancer, studies have yielded varied and inconsistent results, with a few studies showing that there is a significant relationship, while a few others suggested that there is no association between them. A study by Bernhard VR et al. (2019) [78] showed abundant Gram-negative bacteria, particularly *P. gingivalis*, *Fusobacterium* and *Prevotella* species and *T. forsythia,* in the sub-gingival plaque samples. The authors have suggested an indirect mechanism by which periodontitis-induced inflammation might be considered as one of the predisposing factors for breast carcinoma. Sfreddo CS et al. (2017) [79] have found that individuals with periodontitis were two to three times more prone to breast cancer than those who did not have it. However, many other studies found no association between them.

### 3.6. Neurological Disorders

It was noted that marginal periodontitis was linked to Alzheimer’s disease, depression and early intellectual disability [10]. Studies have shown that changes in the cerebral white matter and silent infarctions were higher in individuals with severe periodontitis than in healthy individuals indicating that periodontitis could serve as an important marker for predicting intellectual disability and stroke [10]. In such individuals, due to dental pain and tooth loss, there is decreased masticatory activity with resultant decreased synthesis of acetylcholine which might be responsible for the cognitive impairment [10].

Periodontal pathogens such as *P. gingivalis* and its toxin gingipains [80], *T. denticola* [81] and *C. pneumoniae* [82,83] were shown to be present in patients’ brains with Alzheimer’s disease. Furthermore, it was shown that the levels of gingipains in the brain correlated with neuropathological lesions (neurofibrillary tangles (NFT)) seen in Alzheimer’s disease; thus, suggesting that these pathogens not only release mediators of inflammation, but possess mechanisms to cross the blood–brain barrier and invade the brain. It was noted that in elderly patients with Alzheimer’s disease, a higher level of antibodies against few periodontal pathogens such as *T. forsythia*, *P. gingivalis*, *A. actinomycetemcomitans* [84], *P. intermedia* and *F. nucleatum* [85] were demonstrated than in the healthy group. 

Similar to diabetes, it was also shown that a “bilateral relationship” exists between periodontitis and Alzheimer’s disease. The inflammatory process due to periodontitis was considered as the predisposing factor for the development of Alzheimer’s disease. Increased pro-inflammatory cytokines were demonstrated in elderly patients with both of the diseases [86]. Studies with anti-inflammatory drugs and cytokines have strengthened the opinion that the inflammatory process results in neurodegeneration in Alzheimer’s disease, indicating that nasal NSAIDs could be effectively used in its treatment [87]. Furthermore, fluoxetine was found to be useful not only in clinical depression, but also in suppressing the inflammatory response and severity of periodontal disease [1]. 

### 3.7. Rheumatoid Arthritis (RA)

Both periodontitis and RA are immune-mediated inflammatory diseases with inflammation as a common factor; the common pathobiological processes between the two are very similar in the inflammatory cells involved at the site of inflammation and a variety of inflammatory mediators such as MMP, mediators associated with bone destruction. Studies have revealed that certain periodontal pathogens, specifically *P. gingivalis* and *A. actinomycetemcomitans*, are thought to be associated with citrullination that contributes to the formation of auto-antibodies and altered immunotolerance in an RA susceptible host [88,89]. Some studies have shown that the severity of periodontal disease correlated with the disease activity of RA. Patients with advanced RA are more likely to endure moderate to severe periodontitis as compared to those without RA [90]. Moreover, edentulism and moderate to severe bone loss was more likely in patients with RA than non-RA patients [90]. It was shown that non-invasive periodontal procedures had a positive effect over RA parameters such as ESR, CRP, Disease Activity Score and TNF levels [91].

### 3.8. Fatty Liver

Periodontal pathogens along with their endotoxins and/or cytokines released into the blood circulation can result in endotoxemia, bacteremia and induce an inflammatory response. Hence, periodontitis alone could be considered an independent precipitating factor for nonalcoholic fatty liver disease/nonalcoholic steatohepatitis (NAFLD/NASH) [4,10]. Additionally, the presence of P. gingivalis was significantly higher in NAFLD individuals than in healthy participants [92]. It is worth noting that the infection with type II *P. gingivalis* in an NAFLD mouse model was found to dramatically increase the progression of NAFLD without any other additional treatments such as choline-deficient, l-amino acid-defined diet-fed or LDL receptor knockout [92,93,94]. 

Furthermore, decreased serum albumin levels observed in patients with *P. gingivalis*-positive NAFLD/NASH were suggestive that *P. gingivalis* may be responsible for the reduced liver function with resultant NAPLD or NASH. Interestingly, an appropriate periodontal therapy has been shown to improve the functional parameters of liver such as serum alanine aminotransferase and aspartate aminotransferase in these patients [10]. 

### 3.9. Osteoporosis

Epidemiological studies have also shown a strong association between periodontitis and osteoporosis. More recently, decreased bone mass of femur, tibia and lumbar vertebrae with increased number of bone resorbing cells, inflammatory mediators and type I collagen C-terminal peptide were demonstrated in patients with periodontitis. Additionally, experimental studies with blood samples of rats with periodontitis showed periodontal pathogens such as *F. nucleatum*, *A. actinomycetemcomitans* and *P. gingivalis*. Hence, it was suggested that these organisms through a hematogenous route initiate a systemic inflammatory response with a subsequent reduction in systemic bone density [95].

## 4. Importance of Oral Health

It is well known that poor oral hygiene can lead to the progression or development of many systemic diseases. This breakthrough association between oral and systemic health has led the search for biomarkers in the oral cavity that could assist in the detection of systemic illnesses. The advantages of using the oral cavity as a diagnostic tool are that it is easily accessible, comfortable and allows for non-invasive tests. Therefore, the oral cavity can be used for the early diagnosis and prevention of systemic diseases. 

The link between periodontitis and systemic disease remains the subject of intense research and debate within dentistry. Although the association between periodontitis and non-oral systemic diseases is now better understood, especially with the development of “omics”-based markers for some diseases, the evidence for a causative role is still lacking. Most of the research in this area recommends ‘further studies are needed’, but that should not prevent dental professionals taking a pragmatic approach in promoting a patient’s good oral health to benefit their overall health. It is acknowledged that the gaps in our knowledge remain large [11].

## 5. Conclusions

Periodontal pathogens are mediators of inflammatory reactions in the body; whether they have entered the bloodstream or remained in the oral cavity, they link oral and systemic health. The two-way association between periodontitis and non-oral systemic diseases, with periodontal pathogens being the mediator, needs to be broken down. These findings represent a definite and consistent model in the pathogenesis and treatment of systemic diseases focused on the oral microbiome and antimicrobial-based therapies. Instituting an early and appropriate periodontal therapy, addressing all possible associated precipitating and risk factors may be effective and beneficial in preventing and improving the overall quality of life of the patients. Future studies are anticipated to elucidate the mechanisms through which the periodontal diseases and systemic diseases affect each other.

## Data Availability

The data set used in the current paper will be made available on request from Rakhi Issrani.
